# The value of Institute of Human Virology meeting abstracts and beyond

**DOI:** 10.1186/1742-4690-2-74

**Published:** 2005-12-07

**Authors:** Kuan-Teh Jeang

**Affiliations:** 1 The National Institutes of Health, Bethesda, Maryland, USA

## Abstract

This month *Retrovirology *publishes the meeting abstracts from the 10^th ^annual Institute of Human Virology conference held August 29^th ^to September 2^nd^, 2005 in Baltimore, Maryland, USA. In this editorial, the rationale for publishing meeting abstracts is discussed.

To celebrate the 10^th ^annual meeting of the Institute of Human Virology (IHV), *Retrovirology *publishes this month a supplement [1] which includes 315 abstracts of presentations that took place August 29^th ^to September 2^nd^, 2005 in Baltimore, Maryland, USA. This compilation of meeting abstracts, as with all other items published in *Retrovirology*, will be listed in PubMed, indexed in MedLine, and permanently archived in PubMed Central. The IHV abstracts will be available for all to read in an unrestricted "Open Access" manner. This latter privilege is important because fully two-thirds of the users of free and open data bases such as PubMed are in fact not academics. The users may be patients, students, teachers, or healthcare professionals; and they would be barred from information-access by a fee-based format.

Is there value to publishing Meeting Abstracts? On several levels, the answer appears to be "yes". While the IHV abstracts are the first of its kind for *Retrovirology*, our experience with publishing meeting reports [2-5] tells of strong readership interest. Our statistics show that the published 2004 Cold Spring Harbor Retrovirus Meeting Report [2], a meeting attended by ~500 conferees, has been read in *Retrovirology *3668 times over the past 14 months. A separate report of the 2005 Twelfth West Coast Retrovirus meeting [5], attended by ~125 scientists, was accessed 610 times in the first ten days after its publication. Independently, Scherer *et al*. [6] found in a study of 2,391 meeting abstracts that 51% of the abstracts later appeared as full articles in journals. In another survey, 84% of journals were found to permit the citation of meeting abstracts in bibliographies [7]. Because on average an entire year lags between the time that a paper/poster is presented at a meeting and its eventual publication in a journal, publishing meeting abstracts arguably serves to narrow a knowledge gap between those who attended a meeting and those who did not [7]. Moreover, extant data support that the "open access" approach to publishing scientific information promotes a higher rate of citation to the published work [8]. Thus, it stands to reason that there is value for both authors and readers of *Retrovirology *meeting abstracts.

Let me close this writing by telling you a personal anecdote which illustrated for me why archiving of meeting abstracts is important. In the early 1980's, I was a graduate student working in one of three laboratories worldwide which were competing on the cloning and the characterization of the cytomegalovirus (CMV) immediate-early (IE) promoter. This is the same CMV promoter that is resident in the many mammalian expression vectors which most of you purchase commercially. My memory tells me (although my memory has faded with age) that between 1981 to 1984, I made several presentations on CMV promoter research at the then annual Herpesvirus meeting held at Cold Spring Harbor. Later, in 1990, a patent for the use of the CMV IE promoter was filed by a competitor's group. Many years passed, until approximately five years ago when I unexpectedly received a telephone call from a patent attorney at a high-priced law firm in New York City. The attorney represented a biotech firm which was keen on contesting the issued CMV IE patent. The attorney wanted to know "What did I say publicly about my CMV IE promoter research at meetings?" "And when did I say them?". I recall at that moment when confronted to recall accurately minute historical details critical to a legal contest, I wished fervently for the existence of an open access, easily searchable, repository of meeting abstracts.

*Retrovirology *is committed to the goal of free public access to permanently archived digitally formatted scientific information. Meeting abstracts published in *Retrovirology *are initially viewable online in our journal, and are then permanently deposited into the PubMed Central archive. *Retrovirology *is currently accessed ~1,000 times daily. If you are a meeting organizer interested in the rapid and broad dissemination (with permanent archiving) of the presentations from your conference, it may be worth your while to consider publishing your meeting in *Retrovirology*.

## References

[B1] (2005). Abstracts from the 2005 International Meeting of The Instituteof Human Virology. Retrovirology.

[B2] Freed EO, Ross SR (2004). Retroviruses 2004: Review of the 2004 Cold Spring Harbor Retroviruses conference. Retrovirology.

[B3] Menu E, Müller-Trutwin MC, Pancino G, Saez-Cirion A, Bain C, Inchauspé G, Gras GS, Mabondzo AM, Samri A, Boutboul F, Le Grand R (2005). First Dominique Dormont international conference on "Host-pathogen interactions in chronic infections – viral and host determinants of HCV, HCMV, and HIV infections". Retrovirology.

[B4] Lairmore MD, Fujii M (2005). 12th international conference on human retrovirology: HTLV and related retroviruses. Retrovirology.

[B5] Barry SM, Melar M, Gallay P, Hope TJ (2005). Review of the twelfth West Coast retrovirus meeting. Retrovirology.

[B6] Scherer RW, Dickersin K, Langenberg P (1994). Full publication of results initially presented in abstracts: a meta-analysis. JAMA.

[B7] Kelly JA (1998). Scientific meeting abstracts: significance, access, and trends. Bull Med Libr Assoc.

[B8] Antelman K (2004). Do Open-access articles have a greater research impact?. College Res Libr News.

